# Expression patterns of maize PIP aquaporins in middle or upper leaves correlate with their different physiological responses to drought and mycorrhiza

**DOI:** 10.3389/fpls.2022.1056992

**Published:** 2022-12-15

**Authors:** Ewelina Paluch-Lubawa, Barbara Prosicka, Władysław Polcyn

**Affiliations:** Department of Plant Physiology, Faculty of Biology, Adam Mickiewicz University, Poznań, Poland

**Keywords:** arbuscular mycorrhiza, leaf drought tolerance, plasma membrane aquaporins, *Rhizophagus irregularis*, *Zea mays*

## Abstract

Here we report the effect of *Rhizophagus irregularis* on maize leaf expression of six plasma membrane aquaporin isoforms from PIP1 and PIP2 subfamilies under severe drought development and recovery. The novelty of our study is the finding that leaf-specific mycorrhizal regulation of aquaporins is dependent on the position of the leaf on the shoot and changes in parallel with the rate of photosynthesis and the stomatal response to drought. The transcripts were isolated from the upper third (L3) or ear (L5) leaf, which differed greatly in physiological response to stress within each symbiotic variant. Aquaporins expression in upper L3 leaves appeared to be largely not sensitive to drought, regardless of symbiotic status. In contrast, L5 leaf of non-mycorrhizal plants, showed strong down-regulation of all *PIPs*. Mycorrhiza, however, protected L5 leaf from such limitation, which under maximal stress was manifested by 6-fold and circa 4-fold higher transcripts level for *PIP1s* and *PIP2s*, respectively. Distinct expression patterns of L3 and L5 leaves corresponded to differences in key parameters of leaf homeostasis - stomatal conductance, photosynthetic rates, and accumulation of ABA and SA as phytohormonal indicators of drought stress. In result symbiotic plants showed faster restoration of photosynthetic capability, regardless of leaf position, which we recognize as the hallmark of better stress tolerance. In summary, arbuscular mycorrhiza alleviates short-term drought effects on maize by preventing the down-regulation of plasma membrane aquaporins within middle leaves, thereby affecting stomatal conductance.

## Introduction

One of the evolutionary adaptations that make plants more tolerant to a number of abiotic stresses is symbiosis with arbuscular mycorrhizal fungi (AM), which also refers to the agricultural prospects to improve maize cultivation (Boomsma and Vyn, 2008). AM symbiosis contributes to drought stress alleviation mainly *via* hyphal water and minerals uptake but also by altering expression of some stress related plant genes, including aquaporins (Augé, 2001; Ruiz-Lozano et al, 2012; Bahadur et al., 2019; Cheng et al., 2021).

Although mycorrhiza regulate aquaporin-mediated water flow, the exact symbiotic mechanism is currently not very well understood (Porcel et al., 2006; Aroca et al., 2007; Ruiz-Lozano et al., 2009; Quiroga et al., 2019; Cheng et al., 2021). Plasma membrane aquaporins (PIP) are transmembrane water channels comprised of PIP1 and/or PIP2 isoforms that maintain cell-to-cell flow of water and small solutes according to osmotic gradient (Chaumont and Tyerman, 2014; Verdoucq and Maurel, 2018). Effective water movement across membranes depends on the composition of aquaporin tetramers. When PIP1 isoforms are co-expressed with PIP2s the membrane permeability is increased in comparison to only PIP2 homocomplexes (Fetter et al., 2004; Jozefkowicz et al., 2017). The differences in the expression level of each isoform modify the functionality of PIP1-PIP2 or PIP2-PIP2 tetramers (Boursiac et al., 2005; Fouquet et al., 2008; Alexandersson et al., 2010; Jozefkowicz et al., 2017).

Among AM-mediated mechanisms helping plants to cope with drought situations is the change in hormonal signalization, including abscisic (ABA) and salicylic (SA) acids (Aroca et al., 2008a; Sánchez-Romera et al., 2018). ABA affects plant water status by regulating both root hydraulic conductance (Aroca, 2006; Rosales et al., 2019) and stomatal movements which results in decreased rate of transpiration (Zhang et al., 2006; Wilkinson et al., 2012). SA also mediate in drought tolerance mechanisms by affecting stomatal closure and osmolytes biosynthesis (Miura and Tada, 2014; Li et al., 2017). In addition, SA is a phytohormone strictly related to root colonization by mycorrhizal hyphae (Foo et al., 2013).

Number of studies reported combined effects of ABA and AM on aquaporins activity, generally increasing root water conductance (Aroca et al., 2008a; Aroca et al., 2008b; Ruiz-Lozano et al., 2009; Calvo-Polanco et al., 2019). An opposite effect on root hydraulic conductance has been proposed for SA, suggesting inhibition of cell-to-cell water flow by internalization of specific aquaporins in cell vesicles (Boursiac et al., 2008; Du et al., 2013) or by down-regulation of PIP isoforms of high water conductance (Quiroga et al., 2018).

In spite of driving local transport, aquaporins affect also long distance parameters such as root hydraulic conductivity (Maurel et al., 2008; Zarrouk et al., 2016; Sade and Moshelion, 2017). Plants with established symbiosis have significantly higher water flow by the apoplastic pathway in roots but can also modulate the shift between apoplastic and cell-to-cell transport (Bárzana et al., 2012; Quiroga et al., 2019). It is suggested that changes in ABA and SA levels may contribute to such a switching mechanism by affecting the activity of PIPs (Calvo-Polanco et al., 2014; Sánchez-Romera et al., 2016).

Although several experiments have been conducted in recent years to determine how mycorrhizal fungi regulate maize aquaporins, most of the studies were conducted on the roots (Bárzana et al., 2012; Barzana et al, 2014; Quiroga et al., 2017; Quiroga et al., 2019). Regulation of PIPs in the leaves of mycorrhized maize has not been extensively studied. Considering PIPs involvement in leaf response to drought, the water transport by aquaporins becomes a key component since they allow water movement from vascular bundle to mesophyll tissues even under gradual blockage of apoplastic outflow and by this route may still maintain transpiration stream (Heinen et al., 2009; Maurel et al., 2015; Scoffoni et al., 2017). Furthermore, the contribution of aquaporins to leaf homeostasis may be further enhanced if an alternative hyphal water supply occurs, which may slow down the reduction of mesophyll cells membrane permeability and stomata closure (Bahadur et al., 2019; Cheng et al., 2021).

In our previous study (Polcyn et al., 2019), we established nutritionally non-limiting pot system to examine the response of mycorrhized maize to drought. Similar to the present report, the plants were subjected to progressive water deprivation with the intention of breaking resistance to water loss. As we demonstrated, AM plants significantly faster restored stomatal conductance and photosynthetic rates and recovered from symptoms of drought-induced leaf senescence. Following Augé (2001), we recognized this as a hallmark of better drought tolerance.

Despite the high doses of NPK in our experimental set-up, a targeted distribution of phosphorus or nitrogen to upper or middle leaves was observed, regardless of symbiotic status (Polcyn et al., 2019). We found an analogous preference in the present study, as the photosynthetic efficiency of the upper leaves was shown to be higher than that of the middle leaves.

Such a variation in photosynthesis may be caused by different access to light along the maize shoot. Leaf acclimatization to light is explained by a complex mechanism, including the abundance and activity of leaf aquaporins (Prado and Maurel, 2013; Chaumont and Tyerman, 2014). There is an indication that aquaporins may mediate acclimation to light keeping the turgor of stomatal guard cells (Yaaran and Moshelion, 2016) but also as facilitators of transcellular diffusion of water (Kim and Steudle, 2007) and CO_2_ (Gago et al., 2020) within mesophyll tissues.

After considering the above facts, we set out to answer the question of whether physiological differences between upper and ear leaves are related to the time course of PIP aquaporin expression under progressive drought development and whether the stress-alleviating effect of mycorrhiza is reflected in this co-occurrence. Therefore, the objective of the present study was to determine changes in the gene expression pattern of the PIP1 and PIP2 isoforms in relation to key parameters of leaf homeostasis: stomatal conductance, photosynthetic rates, and accumulation of ABA and SA as phytohormonal indicators of drought stress.

## Material and methods

### Plant culture conditions and sampling

Plant cultures (*Zea mays* L., hybrid Opoka, HR Smolice, Poland) and seedling inoculation with *Rhizophagus irregularis* spores, obtained from monoxenic root cultures (Adholeya et al., 2005) were carried out in a high fertilized pot system, strictly according to the procedure described previously (see Supplementary Data Sheet 1 in Polcyn et al., 2019). To avoid the risk of precipitation of fertilizer salts and obtain precise dosage, we used a soil-free semi-hydroponic setup frequently irrigated with a water-soluble fertilizer. The substrate mix of coconut fiber and sand has excellent water and air exchange properties, allowing to obtain severe but quickly reversible soil drought effects. The culture was continued until silking (63 BBCH stage, 12 weeks after sowing), making sure that long-term hyphae vitality remained undisturbed and that symbiotic plants did not differ from non-mycorrhized counterparts in terms of shoot size and plant nutritional status.

The plants were watered 3 × 200 mL per week. In this volume a fully soluble commercial fertilizer of lowered phosphorus content was included (Kristalon Blue label, Yara Poland). The fertilization level, defined as not detrimental to long-term hyphae vitality, was: 114 mg N/36 mg P_2_O_5_/120 mg K_2_O/18 mg MgO, as expressed in weekly doses per plant (Polcyn et al., 2019). To initiate the progressive drought development, watering was limited to 30% of the full irrigation volume, until of leaves reached <-1.5 MPa. The culture was then rehydrated for 4 days to the levels of the control, non-stressed plants. The experiment was repeated in two independent cultures.

Research was conducted at two stem positions – the 3rd from the top leaf (L3) and the ear leaf (L5), where the light intensity was 720 or 450 µE m^-2^ s^-1^, respectively. The samples for RNA, ABA and SA extraction were taken as a mix of leaf strips collected from six plants of each symbiotic variant, from two independent cultures, cut from the same leaf blades on consecutive days of water treatment.

### Measurements of plant-physiological parameters

The midday leaf water potential, the light-saturated leaf gas exchange capacity, the chlorophyll fluorescence kinetics, and leaf nitrogen management efficiency were measured as described in (Polcyn et al., 2019).

Leaf gas exchange capacity was determined with Q-Box CO650 Plant CO_2_ Analysis Package (Qubit Systems, CA), coupled with an infrared gas analyzer. After 20 min exposition to 3000 μmol m^−2^ s^−1^ photon-flux density, to reach the maximal stomatal opening, the following parameters were calculated: light-saturated photosynthetic rate (Amax, µmol CO_2_ m^−2^ s^−1^) and maximum stomatal conductance to H_2_O (gs, mol H_2_O m^−2^ s^−1^).

The evaluation of leaf nitrogen status was based on chlorophyll/flavonoids ratio (NBI, Nitrogen Balance Index) measured with Dualex 4 Scientific fluorimeter (Force-A, Orsay, France). The readings covered upper surface of apical half of leaves and data was presented as the averaged values of 50 sampling points from four plants for each symbiotic and drought variant.

The chlorophyll fluorescence induction kinetics (the Kautsky effect) were estimated using a pulse amplitude modulated (PAM) fluorimeter (FMS1, Hansatech) to evaluate PSII efficiency in a dark-adapted state (Fracheboud and Leipner, 2003). The fluorometric indices were calculated as follows: Fv/Fm = (Fm-F0)/Fm, maximum PSII quantum yield; ΦPSII = (Fm’-Ft)/Fm’, effective quantum yield of PSII electron transport; NPQ = (Fm-Fm’)/Fm’, steady-state nonphotochemical fluorescence quenching. The symbols are defined as follows: F0 - minimum fluorescence in dark-adapted state; Fm - maximum fluorescence in dark-adapted state; Fm’ - steady-state maximum fluorescence under actinic light; Ft - steady-state fluorescence under continuous light.

### Determination of salicylic acid

Salicylic acid in the free (SA) and conjugated form (as a glucoside, SAG) was extracted from frozen leaf samples (0.5 g) and quantified by HPLC method as described by Yalpani et al. (1993) with minor modifications (Formela-Luboińska et al., 2020). The chromatograph composed of 2699 Separation Module Alliance and the 2475 Multi-λ Fluorescence Detector (Waters Corp., Milford, MA, USA) was used. The content of the salicylic acid released from its glucoside (SAG) was calculated as the difference between assays without and with glucoside enzymatic degradation and expressed in nanograms per gram of fresh weight (ng SA × g^−1^ FW).

### Determination of abscisic acid

The isolation and estimation of abscisic acid (ABA) by HPLC was performed according to Moore (1990) with some modifications (Bandurska and Stroiński, 2003) on frozen leaf samples (0.5 g). ABA-methyl ester was added during homogenization as an internal standard for estimating extraction efficiency. Extracts (20 µL) were resolved in reverse-phase column (Discovery C18, 24 × 4.6 mm, Supelco Inc., Bellefonte, PA, USA) using Varian Star 6.3 Chromatography Workstation (Varian Inc., Walnut Creek, CA, USA). The level of ABA was expressed in micrograms per gram of fresh weight (µg ABA × g^−1^ FW).

### RNA isolation and semi-qPCR conditions

RNA was isolated as described by Murray and Thompson (1980) following DNase treatment with RQ1 RNAse-free DNase (Promega, Leiden, The Netherlands). RNA concentration was assessed spectrophotometrically using NanoDrop ND-1000 (Isogen Life Science, DeMeern, The Netherlands) and further qualified by agarose electrophoresis.

Reverse transcription was performed with 1.5 µg of total RNA using the Revert Aid H minus first strand cDNA synthesis kit (Fermentas, St Leon-Rot, Germany), following the manufacturer’s instructions. cDNA (100 ng) from each sample was used in the semi-qPCR estimations.

cDNA amplifications were carried out using Dream Taq Green DNA Polymerase (Fermentas, St Leon-Rot, Germany). An initial denaturation step was conducted at 95°C for 2 min, each cycle consisted of 30 s at 95°C, 30 s at an annealing temperature depending on the primers combination (see **Supplementary Table 1**), and 60 s elongation at 72°C, followed by a final extension of 10 min at 72°C. To ensure that PCR products were recovered from the linear phase of amplification, the range of linearity between cycle numbers and relative amounts of RT-PCR products was checked in the preliminary experiments (**Supplementary Figure 1**). For further analyses, 35 cycles and 100ng cDNA/µl was chosen.

Gel images were taken with ChemiDoc™ MP Imaging System (Bio-Rad, USA) and ImageLab 5.1 software. The densitometric estimation of transcripts accumulation was conducted using *Gelix One* software (Biostep, Jahnsdorf, Germany), with normalization related to S0 time point, done separately for mycorrhized and non-mycorrhized plants.

The primers for *PIP1* and *PIP2* aquaporins were based on literature data (McDonough and Marbán, 2005), while for the reference genes they were designed using Primer3 (ver. 0.4.0) or Primer-BLAST (www.ncbi.nlm.nih.gov/tools/primer-blast/) software. For normalization purposes, the 18S rRNA and elongation factor-1 (EF-1) genes were tested as the reference (**Supplementary Figure 2**). In final calculations 18S rRNA was used as a reference gene.

To ensure that designed primers flank the target cDNA, the PCR products were identified by sequencing. The PCR products were purified with thermosensitive Exonuclease I and FastAP Alkaline Phosphatase (Thermo Scientific) and sequenced with BigDye Terminator v3.1 on an ABI Prism 3130XL analyzer (Applied Biosystems) according to the manufacturer’s instructions. The precision of the sequence chromatograms was verified using FinchTV1.3.1 (Geospiza Inc.). The similarity searches of coding sequences obtained were performed on the NCBI server (http://www.ncbi.nlm.nih.gov) using NCBI Standard Nucleotide BLAST (Altschul et al., 1990).

### Statistics

The obtained experimental data were presented as means of number of replicates (n) ± SEM. The statistical analysis was conducted using GraphPad Prism 6 (GraphPad Software, Inc., CA, USA) with the Student’s t-test, for groups with normal distribution (Shapiro-Wilk test), one-way or two-way analysis of variance (ANOVA) and Tukey-Kramer multiple comparison test. Statistically significant results were those for which the level of statistical significance value (*p*) reached: *p ≤* 0,05 (*), *p*<0,01 (**) or *p ≤* 0,001 (***).

## Results

### The effect of drought and mycorrhiza on nitrogen management and photochemical PSII efficiency on two leaf positions on the shoot

All physiological, hormonal and molecular characteristics were investigated in the two positions of the shoot (counted from the top) – the upper (L3) or middle (ear) leaves (L5). Before stress imposition, both symbiotic variants did not differ significantly in nitrogen and phosphorus content within each leaf shoot position investigated (not shown). This was due to the high-fertilized cultivation system used, discussed in our previous work (Polcyn et al., 2019), in which the symbiotic state does not affect the size of the shoot and the nutritional status of the plant. Using this cultivation setup, the level of mycorrhizal colonization was achieved in the present study at the similar level as previously, reaching stable, more than 55%, arbuscular abundance in colonized parts of root fragments at the time of experiments (12 weeks after sowing, not shown).

**Figure 1:**
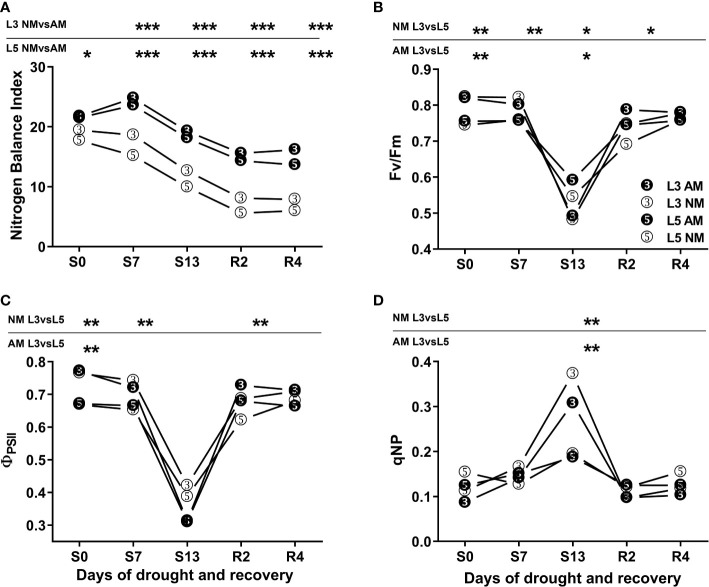
Nitrogen Balance Index (NBI) **(A)** and photochemical efficiency **(B-D)** of upper (L3) and middle (L5) leaves of mycorrhized (AM) or non-mycorrhized (NM) plants during following days of water treatment. The values represent fully irrigated variants (S0), days of drought (S7, S13) and rehydration (R2, R4). ΦPSII – effective quantum yield of PSII electron transport, Fv/Fm - maximum PSII quantum yield potential, qNP – non-photochemical quenching of chlorophyll a fluorescence. Asterisks denote statistically significant differences between AM and NM means (n=50) for NBI values, and between L3 and L5 means (n=6) within each symbiotic variant for photosynthetic indices. Statistics were calculated according to Students t-test (* *p*<0.05, ** *p*<0.01, *** *p*<0.001), standard deviation values were less than 15% and were omitted for clarity.

Both for upper (L3) and middle (L5) leaves the mycorrhized (AM) plants presented higher nitrogen balance index (NBI) at each day of water treatment (**Figure 1A**). We did not observe any significant variation in AM plants between L3 and L5 leaves, whereas for non-mycorrhized (NM) plants statistically significant differences were seen only at 7^th^ day of drought.

Photochemical PSII efficiency was clearly different on the two leaf shoot positions but comparable between symbiotic variants (**Figures 1B–D**). During the first seven days of drought the upper leaves (L3) had higher level of effective quantum yield of PSII electron transport (ΦPSII) or maximum PSII quantum yield potential (Fv/Fm) than middle leaves (L5). However, under the most severe drought that advantage no longer existed due to light stress. It can be inferred from non-photochemical chlorophyll fluorescence quenching (qNP) values were twice higher in L3 than in L5 leaves along with a strong lowering of Fv/Fm index.

**Figure 2:**
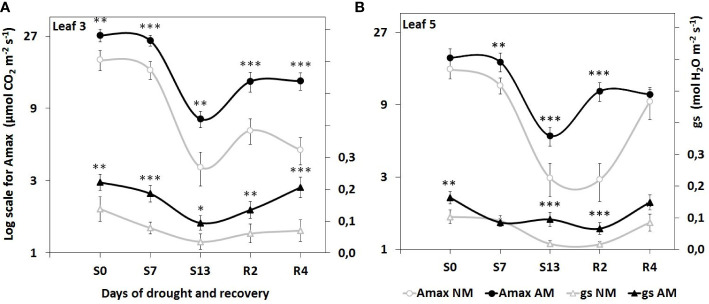
Evaluation of light-saturated leaf gas exchange efficiency in **(A)** apical (L3) and **(B)** middle (L5) leaves, at subsequent time points of drought development (S0-S13) and rehydration (R2, R4), in relation to the presence (AM) or the absence (NM) of mycorrhiza. Amax: light-saturated CO_2_ assimilation rate, gs: maximum stomatal conductance to H_2_O. Points represent mean values and standard deviation. Statistically significant differences between AM and NM means (n=6), calculated using Students *t*-test are presented with asterisks (* *p*<0.05, ** *p*<0.01, *** *p*<0.001).

Consistently higher values of CO_2_ assimilation (Amax) and stomatal conductance (g_s_) in mycorrhized plants through all three phases of water treatment indicated enhanced capability of photosynthesis. Furthermore, these indices were inhibited by drought to a lesser extent than in NM plants (**Figure 2**). Such a detrimental effect was especially distinct at L5 leaf level of NM plants, where we observed low values of gas exchange parameters long-lasting even until the second day of rehydration (R2) in contrast to faster recovery of AM counterparts.

### The effect of drought and mycorrhiza on abscisic acid and salicylic acid accumulation on two leaf levels

**Figure 3:**
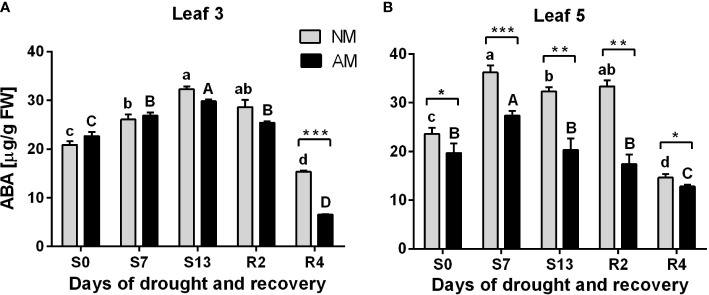
Abscisic acid (ABA) accumulation patterns for L3 **(A)** or L5 **(B)** leaves at subsequent time points of drought development (S0-S13) and rehydration (R2, R4), in relation to the presence (AM) or the absence (NM) of mycorrhiza. Bars represent mean values and standard error of mean (n=6). Statistically significant differences between AM and NM means, calculated using Students *t*-test are presented with asterisks (* *p*<0.05, ** *p*<0.01, *** *p*<0.001). Lower-case letters show significant differences between NM means and upper-case letters significant differences between AM means in following days, calculated using ANOVA test.

Increasing abscisic acid (ABA) accumulation is involved in the mechanism of stomatal closure against abiotic stresses. We observed drought-induced ABA increase both in L3 and L5 leaves (**Figure 3**). This effect was, however, independent of symbiotic status in upper leaf (L3), with exception of the 4^th^ day of recovery (R4) when ABA level in AM plants was lower by 57.3%.

In contrast, a clear difference between mycorrhized and non-mycorrhized plants occurred at L5 leaf level. ABA accumulation was significantly lower for AM plants since the beginning of the experiment and in consecutive days of drought and rehydration. Note that it corresponds with enhancement in leaf gas exchange capability in L5 leaf but not in upper L3 leaf (**Figure 2**) which means that mycorrhization induces less ABA production *per se* and not due to water stress.

**Figure 4:**
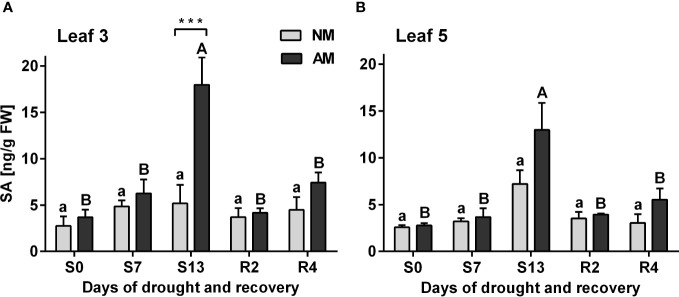
Salicylic acid accumulation patterns for L3 **(A)** or L5 **(B)** leaves at subsequent time points of drought development (S0-S13) and rehydration (R2, R4), in relation to the presence (AM) or the absence (NM) of mycorrhiza. Bars represent mean values and standard error of mean (n=6). Statistically significant differences between AM and NM means, calculated using Students *t*-test are presented with asterisks (*** *p*<0.001). Lower-case letters show significant differences between NM means and upper-case letters significant differences between AM means in following days calculated using ANOVA test.

The drought-dependent accumulation of SA was a hallmark only of AM plants, with a most dramatic effect at the day of the maximum drought (S13). An increase in concentration of this hormone is observed in that day (S13) in L3 and L5 leaves by 247.5% and 79.9%, respectively (**Figure 4**). The statistical difference between symbiotic variants was observed also at this time point, but only for upper leaves (L3).

### Time course analysis of *PIP1* and *PIP2* aquaporins expression patterns

**Figure 5:**
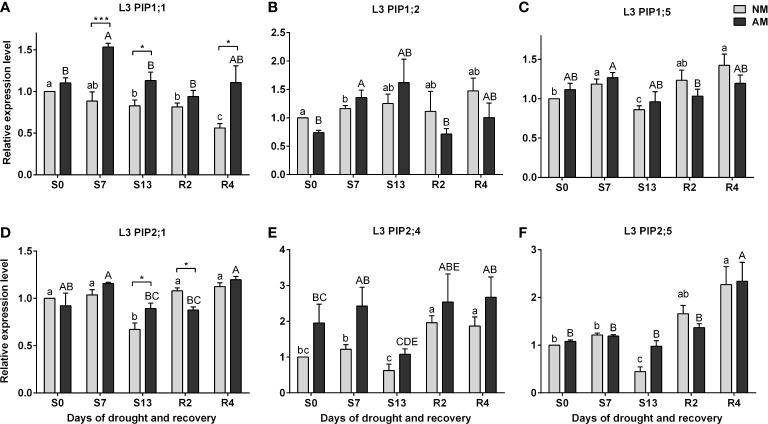
Analysis of aquaporins **(A)** *PIP1;1*, **(B)** *PIP1;2*, **(C)** *PIP1;5*, **(D)** *PIP2;1*, **(E)** *PIP2:4*, **(F)** *PIP2;5* transcipt accumulation in leaf 3 (L3) during the development of drought (S0-S13) and rehydration (R2, R4). The results were normalized to the expression of reference gene *18S RNA* and presented as a relative to S0 time point for NM plants. The bars represent mean values (n=6) and standard error of mean of three technical replicates taken from two independent cultures as extracts from a mix of leaf strips from six plants of each symbiotic variant. The asterisks show statistically significant differences between NM and AM calculated using Student’s *t*-test (* *p*<0.05, *** *p*<0.001). Lower-case letters show significant differences between NM means and upper-case letters significant differences between AM means in following days calculated using ANOVA test.

The efficiency context of leaf physiology and the accumulation patterns of ABA and SA presented above were examined as a background for transcriptional analysis of leaf aquaporins expression (**Figure 5**). For the upper leaves (L3), the expression of aquaporins appeared insensitive to drought, irrespective of symbiotic state, except for transcripts of *PIP2;1* and *PIP2;5* which accumulation decreased under maximal stress in relation to normal watering (S0 vs S13 time points) (**Figures 5D, F**). The only variation between symbiotic variants in L3 leaf was observed for *PIP1;1* and *PIP2;1* transcripts, with higher accumulation for mycorrhized plants during the days of drought (**Figures 5A, D**).

**Figure 6:**
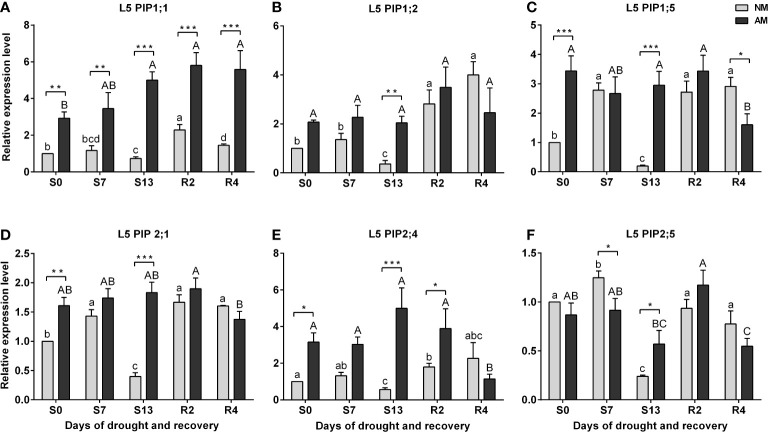
Analysis of aquaporins **(A)** *PIP1;1*, **(B)** *PIP1;2*, **(C)** *PIP1;5*, **(D)** *PIP2;1*, **(E)** *PIP2:4*, **(F)** *PIP2;5* transcipt accumulation in leaf 5 (L5) during the development of drought (S0-S13) and rehydration (R2, R4). The results were normalized to the expression of reference gene *18S RNA* and presented as a relative to S0 time point for NM plants. The bars represent mean values (n=6) and standard error of mean of three technical replicates taken from two independent cultures as extracts from a mix of leaf strips from six plants of each symbiotic variant. The asterisks show statistically significant differences between NM and AM calculated using Student’s *t*-test (* *p*<0.05, ** *p*<0.01, *** *p*<0.001). Lower-case letters show significant differences between NM means and upper-case letters significant differences between AM means in following days calculated using ANOVA test.

In the case of ear leaves (L5) a strong but temporary inhibition of both *PIP1s* and *PIP2s* expression at the maximum drought (S13) was the hallmark of non-mycorrhized plants, in contrast to very stable expression of these aquaporins in AM counterparts (**Figure 6**). Increased levels of transcript accumulation in the L5 leaf of AM plants also occurred during the optimal watering phase prior to the onset of stress, but except for *PIP2;5* (**Figure 6F**).

**Figure 7:**
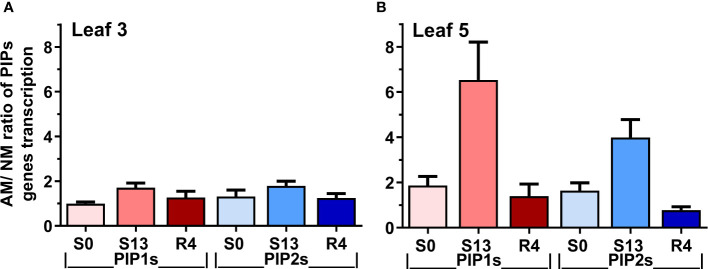
Expression ratio of mycorrhized (AM) to non-mycorrhized (NM) plants for *PIP1* or *PIP2* genes at **(A)** upper (leaf 3) or **(B)** middle (leaf 5) shoot levels. The indexes were calculated for optimal irrigation (S0), the maximum drought (S13) and rehydration (R4) by dividing the total accumulation of aquaporin transcripts of PIP1 or PIP2 aquaporins. Bars represent mean values and standard error of mean. The calculations are based on mean values of AM and NM plants, taken from **Figures 5**, **6**.

The **Figure 7** summarizes the ratio of the averaged aquaporins expression level of mycorrhized to non-mycorrhized plants for upper or middle leaves. The calculated indices show much larger differences in expression between AM and NM plants in leaf 5 than in leaf 3. A particularly large difference in aquaporin expression in favor of AM plants was observed for leaf 5 on the day of maximum drought (S13), when 6-fold more *PIP1s* transcripts accumulated, while the prevalence of *PIP2* genes expression appeared to be 4-fold.

## Discussion

### The question of leaf aquaporins participation in mycorrhiza-assisted drought tolerance mechanisms

The relationship between mycorrhizal water flux and increased stomatal water conductance was confirmed for many species, which has recently been evaluated by meta-analysis (Augé et al., 2015). In the present study, a greater ability of mycorrhized plants to recover leaf gas exchange parameters (Amax, g_s_) after drought was also shown (**Figure 2**). To explore the molecular components of this effect we examined the expression patterns of leaf plasma membrane aquaporins (PIP) under different plant symbiotic status and water treatments with relation to key parameters of leaf homeostasis - stomatal conductance, photosynthetic rates, and accumulation of ABA and SA as phytohormonal indicators of drought stress.

Although plasma membrane aquaporins drive local, cell-to-cell water (and other solutes) transport they apparently participate also in maintaining whole plant hydraulic conductivity and leaf transpiration (Ruiz-Lozano et al, 2006; Aroca et al., 2008b; Ruiz-Lozano et al, 2009). Recent hypothesis suggests a causal relationship between drought-regulated expression of root PIPs and intensity of long-distance water transport (Aroca et al., 2012; Moshelion et al., 2015; Maurel et al., 2016; Sade and Moshelion, 2017). Nevertheless, little is known about the mycorrhizal regulation of PIPs contribution to lateral water transport within leaves.

PIP transcripts were isolated from the upper third (L3) or ear (L5) leaves, differing greatly in physiological response to stress within each symbiotic variant but also in the access to light. The novelty of our study is the finding that leaf-specific AM regulation of aquaporins depends on the position of the leaf shoot and changes in parallel with the rate of photosynthesis and the stomatal response to drought.

### Leaf physiology and hormonal signalization respond differently to drought with respect to leaf position on the shoot and plant symbiotic status

The severity of drought stress was evaluated by measuring photochemical PSII efficiency, CO_2_ assimilation rate, stomatal conductance, nitrogen status and hormonal signaling within leaves (**Figures 1**-**4**). Detailed information gathered from these parameters, allowed us to define three phases of leaf stress (progressive, severe but recoverable, and fast recovery).

Nitrogen loss under given stress conditions was monitored by fluorimetry using highly correlated nitrogen balance index (NBI), evaluated previously as a non-destructive indicator of drought-induced leaf senescence (Polcyn et al., 2019). During the drought progression and recovery, we did not observe any significant variation of NBI values between L3 and L5 leaves within each symbiotic variant (**Figure 1A**). However, higher NBI values of AM plants over all water treatments pointed to increased efficiency of leaf nitrogen management due to mycorrhiza.

Previously, analyzing the effect of *Rhizophagus irregularis* on maize physiology under different fertilization levels, we found that leaves of each shoot position differ significantly in phosphorus and nitrogen assimilation efficiency (Polcyn et al., 2019). It is not a surprise in case of such a tall plant, that it maintains better physiological condition of the upper leaves in cost of the lower shoot levels. It might be a result of redirecting the resources from older leaves, which undergone faster senescence in unfavorable field conditions and have become the donor organs for younger leaves (Miersch et al., 2000; Hörtensteiner, 2009). This effect could also be driven by shading by other leaves, especially under phytotron light sources. In the present study we testified physiological condition of leaves on the two shoot positions of the mature maize. In our growth cabinet, the ear leaf (L5) has access to light of 450 μE m^-2^s^-1^ intensity whereas the photon densities at the upper leaves (L3) were as high as 720 μE m^-2^s^-1^, hence their photo-dependent processes could run with better efficiency.

The chlorophyll fluorescence quenching analysis showed a clear difference between the two above-mentioned leaf positions, with L3 leaf having more efficient PSII photochemistry during the first days of water treatment (**Figures 1B, C**). Nevertheless, under severe drought upper leaves showed symptoms of light stress regardless of the symbiotic state of the plants (**Figure 1D**).

Stomata closure is a mechanism protecting plants from water loss during drought, which results in lowering the parameters of gas exchange. This dependency was also observed in our study (**Figure 2**). Consistently higher light-saturated gas exchange efficiency of AM plants through all three phases of water treatment indicated enhanced photosynthetic capability of mycorrhiza-supported plants, however in cost of water loss risk.

The hormonal data agreed with differences in dynamics of stomata closure and photosynthetic deprivation between L3 and L5 leaves. It also confirmed the less severe stress within AM plants and much shorter time needed to enter recovery from the stress (**Figure 2**).

Abscisic acid (ABA) accumulation acts as a signal of soil water deprivation leading to stomata closure (Wilkinson and Davies, 2002). Considering this, we could point for the upper leaves at 13^th^ day of drought treatment as the moment when the stress was maximal but recoverable. We could also notice that ABA pattern did not differ between symbiotic counterparts. In contrast, the intensity of ABA signal within ear leaf raised to the highest level much faster – at 7^th^ day of drought. However, much lower ABA accumulation suggested that the stress on this shoot position was apparently less heavy in AM supported plants (**Figure 3**).

Salicylic acid (SA) is not only the key signal of biotic stress, but also an important regulator of photosynthesis and stomata closure also against abiotic environmental factors (Melotto et al., 2006; Khan et al., 2015). Very high accumulation of endogenous SA at the maximum drought, shown in AM but not in NM plants might play such a function (**Figure 4**).

It is worth noting that the regulation of water conductance by both ABA and SA may be related to root aquaporins expression pattern, which has also been shown in AM plants (Porcel et al., 2006; Ruiz-Lozano et al, 2006; Quiroga et al., 2018). Parent et al. (2009) demonstrated that ABA caused upregulation of genes and protein abundance of most of the PIP isoforms and never caused their decrease. It is coherent with results of Jang et al. (2004) obtained for *Arabidopsis* roots in which exogenous ABA caused an up-regulation of twelve PIP genes. A hypothesis also exists linking SA to aquaporin accumulation, as both endogenous and exogenous SA disturb constitutive endocytosis and may therefore interfere with aquaporins retention in plasma membrane during stress (Boursiac et al., 2008; Du et al., 2013).

All the above-mentioned consideration allowed us to address the question of whether drought-dependent physiological differences between upper and ear leaves arerelated to the time-course of *PIP* aquaporin expression and whether the stress-alleviating effect of mycorrhiza is reflected in the co-occurrence patterns.

### Mycorrhizae differentiate the pattern of *PIPs* expression within the ear leaf but not in leaf of upper level

High level of aquaporins may preserve the water transport, thus keeping higher values of stomata conductance and CO_2_ assimilation in mycorrhized plants (Aroca et al., 2012; Moshelion et al., 2015; Maurel et al., 2016; Sade and Moshelion, 2017). Like physiological and hormonal data shown above, the effect of mycorrhiza on aquaporins expression was remarkable mainly for the ear leaf (L5) and was found already under full hydration (**Figure 6**). In contrast, within the upper leaves (L3) this difference was not present (**Figure 5**). Similar situation was observed during the time course of water deficiency since symbiotic effect was remarkable almost only in L5 leaf (**Figure 6**). This was mainly manifested in drastic decrease in transcripts accumulation at the time point of severe stress (S13). Hence, we could distinguish two patterns of regulation, indicating sensitivity or insensitivity of *PIPs* expression to severe water deficiency, which was mycorrhiza-dependent only within ear leaf (**Figure 7**, **Table 1**)

**Table 1:** Sensitivity of *PIP* aquaporin expression to drought in upper leaf (L3) and ear leaf (L5) in relation to plant symbiotic status.

	Sensitivity to drought	Relation to mycorrhiza	PIP aquaporin isoforms
Leaf 3	insensitive	AM	all *PIPs*
	insensitive	NM	all except *PIP2;1* and *PIP2;5*
Leaf 5	insensitive	AM	all *PIPs*
	sensitive	NM	all *PIPs*

Indicated for maximal stress as a significant decrease in relation to normal watering (S0 vs S13 time points), according to data taken from **Figures 5**, **6**.

By averaging this data, we can conclude that in upper leaf the expression of both *PIP1s* and *PIP2s* showed low sensitivity to drought irrespectively of symbiotic status, while in the middle leaf (L5) it was insensitive only for mycorrhized plants (**Table 1**). The regulation of the *PIP2;4* and *PIP2;5* isoforms was more complex. We believe that such different patterns reflect a link with a more effective restoration of L5 physiology after drought in mycorrhizal-assisted plants. It is worth to note that increased levels of transcript accumulation in the ear leaf of AM plants also occurred during the optimal watering phase prior to the onset of stress, but except for *PIP2;5* (**Figure 6**).

As **Figure 7** shows, the effect of mycorrhiza was particularly striking within L5 leaf where *PIP1s* and *PIP2s* expression reached respectively 6-fold and 4-fold higher level under the maximum drought. Considering that PIP1 isoforms are necessary to increase cell membrane permeability (Fetter et al., 2004; Jozefkowicz et al., 2017), it points to a kind of risk-taking mechanism of AM plants, which, together with a greater disposition to open the stomata and carry transpiration at the time of recovery, can be seen as the hallmark of mycorrhiza-improved stress tolerance mechanism.

Development of the drought causes gradual blockage of water transport system in plants, which by the transverse pathway in root as well as in leaf mesophyll is to a large extent, passively subjected to osmotic and transpiration forces. Plant reacts, among others by developing occlusion blocking apoplastic efflux of water from the root bark to soil (Casparian bands are permanent barrier), and on the leaf side lowering of turgor triggers stomatal closure (Yaaran and Moshelion, 2016; Martin-StPaul et al, 2017). During the drought stress, the cell-to-cell water flow by means of aquaporins becomes a key factor in unblocking the water transport from the vascular bundles to root and leaf tissues. Nevertheless, the presence of alternative mycelium water transport system, may supply both the root and leaf aquaporin pathways and in consequence the stomata may be subjected to minor limitations from hormonal signals. Such a conclusion can be drawn for middle leaf (L5) from the consistently higher stomatal conductance throughout whole water treatments (**Figure 2B**) but also from not reduced aquaporin expression at the maximum drought (**Figure 6**, **Table 1**).

The results for the two selected leaf positions show a striking parallel between patterns of ABA and SA accumulation, leaf gas exchange parameters and the expression of aquaporins, all of them appear to maintain leaf homeostasis under drought conditions.

In conclusion, as we have shown in the present study, mycorrhization alters the expression of several leaf-specific aquaporins, however, in a manner dependent on the position on the shoot. This regulation was accompanied by a parallel change in stomatal conductance, photosynthetic rates, and accumulation of ABA and SA as phytohormonal indicators of drought stress. All that shows that some aquaporins might be a key regulators of leaf homeostasis.

**Figure 8:**
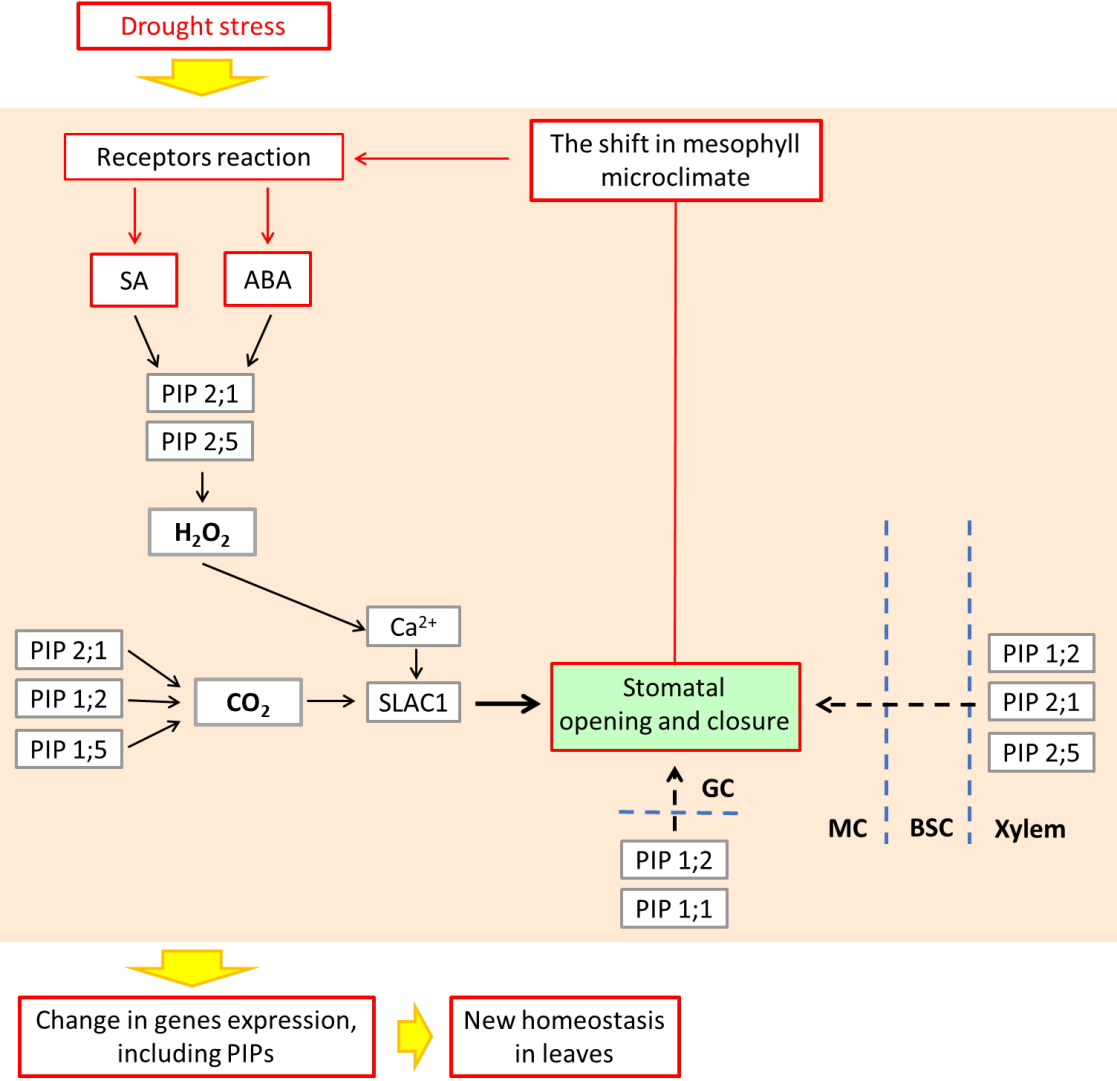
Acclimation to abiotic stress by cyclic regulation of stomata, involving hypothetical role of ABA, SA as the signaling molecules, and leaf PIP aquaporins, leading to microclimate stabilization within the leaf mesophyll. Conceptualized according to Joudoi et al. (2013) and Mittler and Blumwald (2015). ABA and SA control the expression and activity of PIPs which facilitate the movement of water across the membrane of guard cells (GC). In addition to water, aquaporins may also transport small molecules such as CO_2_ and H_2_O_2_, depending on ABA and SA signaling. The maize PIP2;1 together with PIP1;2 and PIP 1;5 are known as the facilitators of CO_2_ entry into GC causing stomatal closure (Ding et al., 2020). PIP2;1 and PIP2;5 also facilitate the ABA-triggered movement of H_2_O_2_that acts in guard cells as Ca^2+^ channels regulator, leading to regulation of stomatal movement. Lastly, PIP1;2, PIP2;1 and PIP2;5 control the influx of water from the xylem, through bundle sheath cells (BSC) to the mesophyll cells (MC) (Heinen et al., 2009; Yaaran and Moshelion, 2016), which completes the picture of aquaporins, maintaining the mesophyll microclimate. ABA, abscisic acid; SA, salicylic acid; SLAC1, slow anion channel; BSC, bundle sheath cells; GC, guard cells; MC, mesophyll cells.

The results of ours and other authors can be placed in a scheme of relationships between aquaporins expression and hormonal, hydraulic, ROS and CO_2_ signals, and resulting cyclic regulation of the stomatal aperture as the elements of leaf mesophyll acclimatization to changing water conditions. An interpretation of these relationships is proposed in **Figure 8** as a feedback loop mechanism that governs water homeostasis in the mesophyll tissues.

Firstly, ABA and SA are components of a crosstalk with other phytohormones such as cytokinins, auxins and gibberellins under stress conditions (Munné-Bosch and Müller, 2013). Recent work by other authors suggests that the expression and activity of leaf PIPs are under complex hormonal mechanisms, including ABA and SA, which are particularly affected during mycorrhizal symbiosis (Chitarra et al., 2016; Ruiz-Lozano et al., 2022).

Secondly, stomatal closure or opening depends on the adjustment of turgor pressure which is facilitated by the movement of water across the membrane of guard cells, which might be facilitated by plasma membrane aquaporins (Heinen et al., 2009; Yaaran & Moshelion, 2016). In addition to water, aquaporins may also transport small molecules such as CO_2_ and H_2_O_2_, the mechanism dependent on ABA and SA signaling (Boursiac et al., 2008; Ding and Chaumont, 2020). The maize PIP2;1 together with PIP1;2 and PIP1;5 are known as the facilitators of CO_2_ entry into guard cell causing stomatal closure (Ding and Chaumont, 2020). The same PIP2;1 and PIP2;5 also facilitates the ABA-triggered movement of H_2_O_2_that acts in guard cells as Ca^2+^ channels regulator, leading to regulation of stomatal movement (Ding and Chaumont, 2020; Ding et al, 2022).

Thirdly, the presence of apoplastic barriers in bundle sheath forces the water to cross the plasma membrane of the xylem-adjacent cells. It was recently shown that several plasma membrane aquaporins: PIP1;2, PIP2;1 and PIP2;5 control the influx of water from the xylem, through bundle sheath cells to the mesophyll (Heinen et al., 2009; Yaaran and Moshelion, 2016), which completes the picture of aquaporins maintaining the mesophyll microclimate.

## Data availability statement

The original contributions presented in the study are included in the article/**Supplementary Material**. Further inquiries can be directed to the corresponding authors.

## Author contributions

EP-L and WP conceived the original research plan and designed the experiments. EP-L and BP performed the experiments. EP-L and WP analyzed the data. EP-L created the figures and tables. EP-L was responsible for writing first draft. WP was responsible for reviewing and editing. All authors contributed to the article and approved the submitted version.
