# Corrigendum: Expression patterns of maize PIP aquaporins in middle or upper leaves correlate with their different physiological responses to drought and mycorrhiza

**DOI:** 10.3389/fpls.2024.1459016

**Published:** 2024-07-15

**Authors:** Ewelina Paluch-Lubawa, Barbara Prosicka, Władysław Polcyn

**Affiliations:** Department of Plant Physiology, Faculty of Biology, Adam Mickiewicz University, Poznań, Poland

**Keywords:** arbuscular mycorrhiza, leaf drought tolerance, plasma membrane aquaporins, *Rhizophagus irregularis*, *Zea mays*

**Text Correction**

In the published article, there was an error.

A correction has been made to **Acknowledgments** which previously stated:

Acknowledgments

We would like to thank Tamara Chadzinikolau for performing the ABA and SA measurements. We would also like to thank Dr. Jan Lubawy, Department of Animal Physiology and Developmental Biology, AMU Poznan, for helping in the statistical data analysis.

The corrected statement appears below:

Acknowledgments

We would like to thank Dr. Tamara Chadzinikolau, Department of Plant Physiology, Faculty of Agriculture, Horticulture and Biotechnology, Poznań University of Life Sciences, Poznan for performing the ABA and SA measurements and developing the method. We would also like to thank Dr. Jan Lubawy, Department of Animal Physiology and Developmental Biology, Adam Mickiewicz University, Poznan, for helping in the statistical data analysis.

The authors apologize for this error and state that this does not change the scientific conclusions of the article in any way. The original article has been updated.

