# Supplementary material for: Expression patterns of maize PIP aquaporins in middle or upper leaves correlate with their different physiological responses to drought and mycorrhiza
Source: Front Plant Sci. 2022 Dec 15;13:1056992. doi: 10.3389/fpls.2022.1056992 (PMC9798212; doi:10.3389/fpls.2022.1056992)
Supplement: Supplementary file 1 [file DataSheet_1.docx]

**Supplementary Information for:**

**Expression patterns of maize PIP aquaporins in middle or upper leaves correlate with their different physiological responses to drought and mycorrhiza**

Ewelina Paluch-Lubawa*, Barbara Prosicka, Władysław Polcyn*

Department of Plant Physiology, Faculty of Biology, Adam Mickiewicz University, Poznań, Poland,

*Correspondence: Ewelina Paluch-Lubawa [e.paluch@amu.edu.pl](mailto:e.paluch@amu.edu.pl), Władysław Polcyn [polcyn@amu.edu.pl](mailto:polcyn@amu.edu.pl)


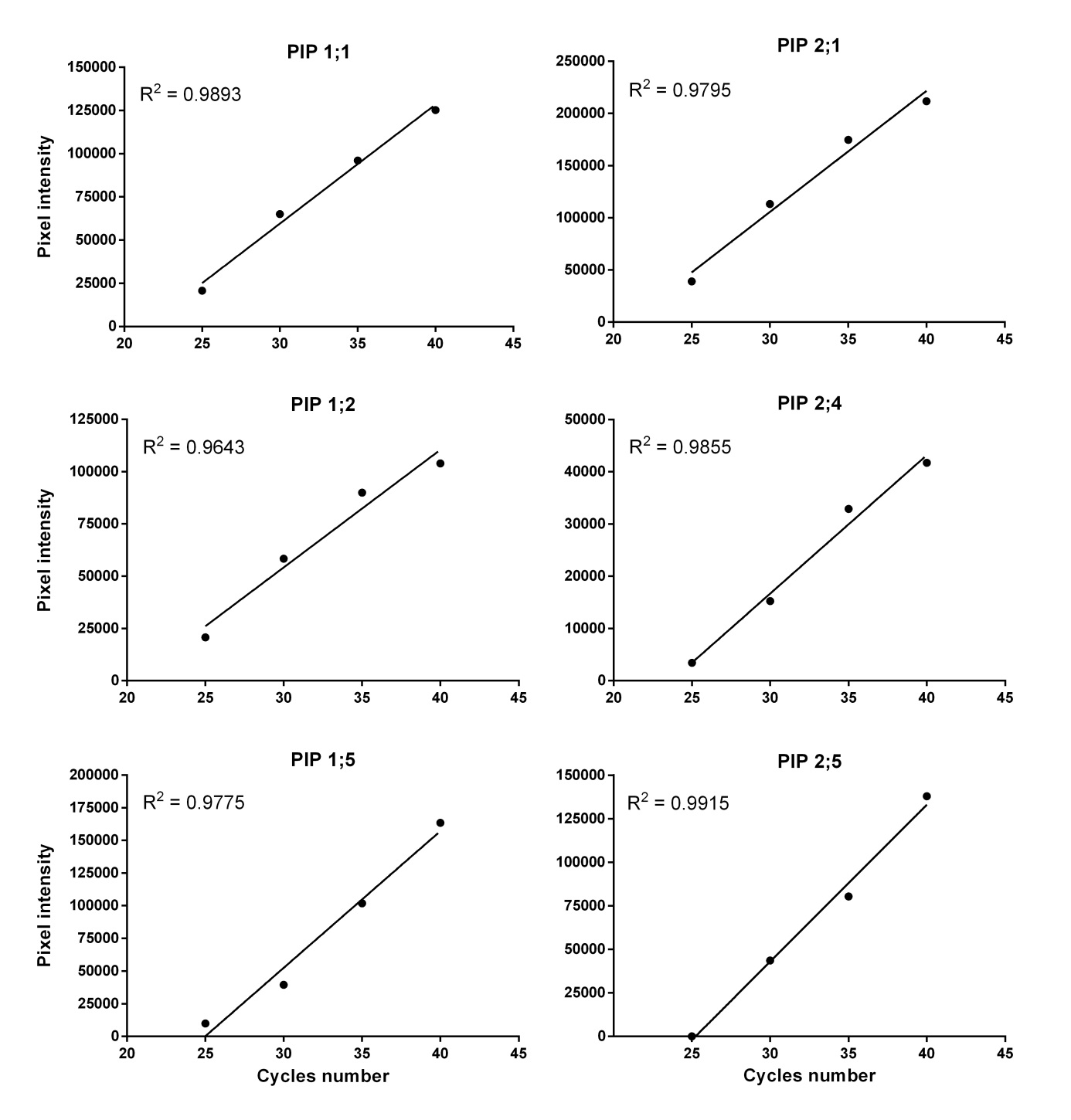


**Supplementary Fig. 1.** Linearity between cycle numbers and relative amounts of RT-PCR products for aquaporins genes.


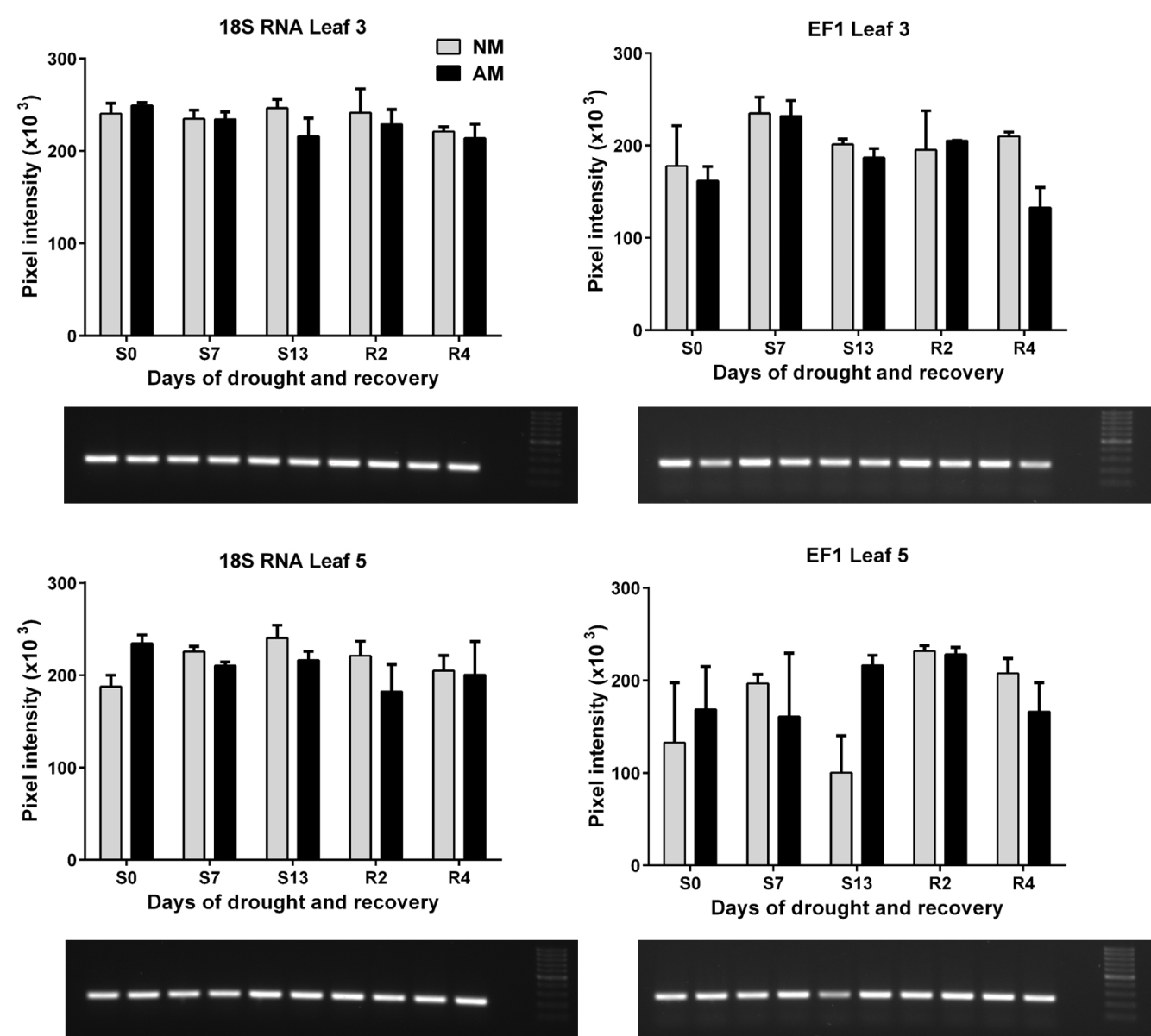


Supplementary Fig.2. Reference genes selected for normlization of transcript expression of aquaporin isoforms. On the left *18S RNA* for leaf 3 and leaf 5, on the right *EF1* for leaf 3 and leaf 5. Electrophorograms proving stability of reference genes are presented below the graphs.

**Supplementary Tab. 1.** A list of the primers for plasma membrane intrinsic proteins (PIPs) genes. Gene name, the primer sequences, and the annealing temperatures used in this study for the semi-qPCR purpose.

| **Gene** | **Forward primer** | **Reverse primer** | **T_M_ [°C]** |
| --- | --- | --- | --- |
| *PIP 1.1* | 5’ TAAAGGAGCCGATGCTGCTG 3’ | 5’ GGATGAACTCTTAAAGCTTGAC 3’ | 52.5 |
| *PIP 1.2* | 5’ GCGTCTTCCTGTGATGTCTTCT 3’ | 5’ AAATCAAGAAAACCCTGAATCG 3’ | 53.5 |
| *PIP 1.5* | 5’ ATTACCAACAGCAACCATGCAG 3’ | 5’ CTTCACCGTACCAAAACCCAAG 3’ | 59.0 |
| *PIP 2.1* | 5’ CGGCCTTCTACCACCAGTACAT 3’ | 5’ CATGATTACATTGCAGGGGAAC 3’ | 59.0 |
| *PIP 2.4* | 5’ CTACCGGAGCAACGCCTAA 3’ | 5’ ATCGGATAAAAACTCACGCAAT 3’ | 53.5 |
| *PIP 2.5* | 5’ GCGCTGCTGTCATCTACAACAA 3’ | 5’ GCAAGCAAAATGCAGTGGAAAT 3’ | 59.0 |
| *EF1* | 5’ GCGGTCATTCAAGTATGCGT 3’ | 5’ TACCAGCCTCAAAACCACCA 3’ | 50.7 |
| *18s RNA* | 5’ TCTTGATTCTATGGGTGGTG 3’ | 5’ GAACATCTAAGGGCATCACA 3’ | 54.0 |
